# Regulation of the antigen presentation machinery in cancer and its implication for immune surveillance

**DOI:** 10.1042/BST20210961

**Published:** 2022-03-28

**Authors:** Adithya Balasubramanian, Thomas John, Marie-Liesse Asselin-Labat

**Affiliations:** 1 Personalised Oncology Division, The Walter and Eliza Hall Institute of Medical Research, Parkville, Australia; 2 Department of Medical Biology, The University of Melbourne, Parkville, Australia; 3 Department of Medical Oncology, Peter MacCallum Cancer Centre, Parkville, Australia

**Keywords:** cancer, immune response, major histocompatibility complex

## Abstract

Evading immune destruction is one of the hallmarks of cancer. A key mechanism of immune evasion deployed by tumour cells is to reduce neoantigen presentation through down-regulation of the antigen presentation machinery. MHC-I and MHC-II proteins are key components of the antigen presentation machinery responsible for neoantigen presentation to CD8^+^ and CD4^+^ T lymphocytes, respectively. Their expression in tumour cells is modulated by a complex interplay of genomic, transcriptomic and post translational factors involving multiple intracellular antigen processing pathways. Ongoing research investigates mechanisms invoked by cancer cells to abrogate MHC-I expression and attenuate anti-tumour CD8^+^ cytotoxic T cell response. The discovery of MHC-II on tumour cells has been less characterized. However, this finding has triggered further interest in utilising tumour-specific MHC-II to harness sustained anti-tumour immunity through the activation of CD4^+^ T helper cells. Tumour-specific expression of MHC-I and MHC-II has been associated with improved patient survival in most clinical studies. Thus, their reactivation represents an attractive way to unleash anti-tumour immunity. This review provides a comprehensive overview of physiologically conserved or novel mechanisms utilised by tumour cells to reduce MHC-I or MHC-II expression. It outlines current approaches employed at the preclinical and clinical trial interface towards reversing these processes in order to improve response to immunotherapy and survival outcomes for patients with cancer.

## Introduction

The advent of immunotherapeutics has revolutionised treatment in cancer. These agents harness our immune system to promote anti-tumour responses and herald the potential for long-term survival in patients with otherwise incurable disease [1]. Specifically, immune checkpoint inhibitors (ICIs) are now standard of care in many solid organ cancers. They block inhibitory signals expressed by either tumour or immune cells, unleashing the brakes on our adaptive immune system to fight cancer cells. Yet only a minority of patients respond [2]. Ongoing research focuses on tumour resistance mechanisms against ICIs. One method of ‘immune escape’ invoked by tumour cells is through alterations of their antigen presentation machinery (APM) [3], making them invisible to the adaptive immune system. The major proteins in the APM are Major Histocompatibility Complex class I and II (MHC-I and MHC-II) and associated subunits (such as Beta 2 Microglobulin [B2M]) [4,5]. Tumour recognition by immune cells requires presentation of non-self peptides (neoantigens) by tumour cells through MHC Class I or II complexes. Loss or reduced expression of MHC or their subunits abrogates T cell-mediated anti-tumour immunity. Defects in MHC expression has been observed in most common cancers at variable frequencies from 0% to 93% [4]. Deciphering mechanisms to reactivate MHC expression by tumour cells may therefore lead to the identification of alternative approaches to increase anti-tumour immunity. This review describes known mechanisms controlling MHC I and II expression in cancer and highlight how these mechanisms could be tackled towards treatment response and improving patient survival.

## MHC-I

### MHC-I function and antigen processing pathway

MHC I molecules encoded by human leukocyte antigen (*HLA*) genes [6] are present on the cell surface of all nucleated cells [7]. They play an evolutionary role in immunosurveillance by presenting intracellular peptides to cytotoxic CD8^+^ T cells. Immunogenic foreign peptides, such as neoantigens, are recognised by T cell receptors (TCRs) on CD8^+^ T cells resulting in cell killing.

The processing of neoantigens is mediated by the ubiquitin-proteasome system [8]. Proteasomes break down endogenous proteins tagged by ubiquitin into oligopeptides (8–13 amino-acid length) to enable effective presentation by MHC-I. Tumour cells that are exposed to oxidative stress or inflammatory stimuli up-regulate immunoproteasomes [9]. These immunoproteasomes have distinct catalytic activity to specifically generate diverse non-self peptides. The cleaved peptides are then transported to the endoplasmic reticulum (ER) by the specialised TAP (transporter associated with antigen processing) protein [10], where they bind to newly synthesised MHC-I molecules. The neoantigen-MHC I complex is released from the ER and then exocytosed into the plasma membrane for presentation to CD8^+^ T cells.

### Immune evasion through down-regulation of MHC-I in cancer

**Figure 1.**
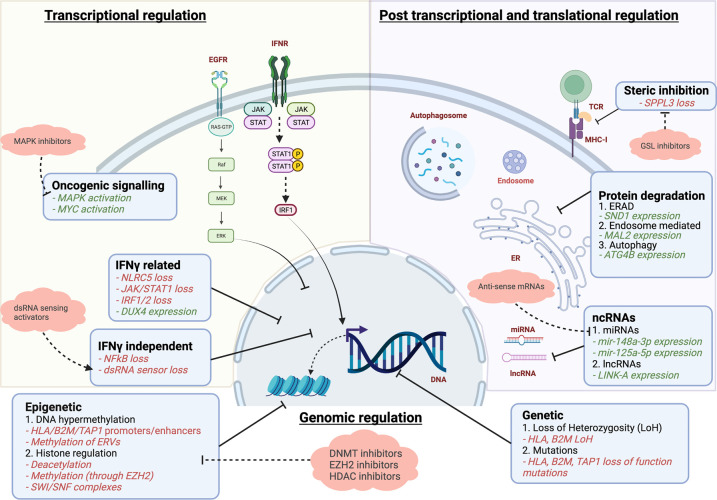
Mechanisms of down-regulation of MHC-I antigen processing pathway by tumour cells and possible therapeutic targets. Green text: expression/over-expression negatively regulates antigen presentation. Red text: reduced/loss of expression negatively regulates antigen presentation.

Immune evasion is a hallmark of cancer to reduce visibility of tumour cells to the immune system[11]. Tumour immune surveillance not only relies on the expression of neoantigens by tumour cells but also the proficient neoantigen presentation to T cells through the MHC complexes. Defects in MHC-I synthesis, transport and loading of appropriate peptides result in low or absent cell surface expression of MHC-I and thus immune evasion. Altered MHC expression can be mediated through *HLA* or *B2M* loss of heterozygosity (LOH) [12]. However recent studies have also shown that loss of MHC protein expression can occur in *HLA* or *B2M* wild-type tumour cells, highlighting the role of non-genetic mechanisms in regulating MHC-I expression [4,13–15] (Figure 1). MHC-I protein loss on immunohistochemistry has been described in almost all types of solid organ tumours. Some studies describing this occurrence in >90% of their cohorts [4].

### Genetic mechanisms of MHC-I loss

#### Loss of heterozygosity

The genes encoding MHC-I are composed of the highly polymorphic class Ia ‘classical’ human leukocyte antigen genes (*HLA-A*, -*B* and -*C*) on chromosome 6 [16]. Loss of both *HLA* alleles results in total elimination of MHC-I expression [17]. Deletions of one allele (*HLA* LOH), reduces MHC-I expression by half. Tumours leverage the genomic instability associated with LOH, whereby a further mutation in the other allele results in complete MHC-I loss, to evade immune recognition [3,18]. This phenomenon has also been described for the gene encoding the B2M light chain and appears to be more prevalent in metastatic compared with primary lesions [19]. A pan-cancer analysis of 83 644 patient samples representing 59 different solid organ tumours revealed the prevalence of *HLA* LOH to be 17% [20]. In Non-Small Cell Lung Cancer (NSCLC), *HLA* LOH has been shown to be both a negative prognostic and predictive biomarker for ICI therapy [21].

#### Somatic mutations

Somatic mutations in *HLA* alleles may also have similar functional implications to deletions, precluding effective neoantigen presentation [22]. Whole-exome sequencing (WES) analysis of a TCGA cohort involving 7930 paired tumour and healthy samples revealed the presence of 298 non-silent mutations in 3.3% of patients [23]. HLA mutations were more prevalent in head and neck, lung squamous and stomach cancers [23]. These findings support earlier work demonstrating the presence of *HLA* mutations and other components of the MHC-I APM pathway including *TAP1* in small cell lung cancer (SCLC) and melanoma cell lines, and *B2M* in human melanoma tumours [24–26].

### Epigenetic silencing

#### DNA hypermethylation

Hypermethylation of gene promoters and enhancers of *HLA* alleles, *B2M* and other APM regulatory genes have been described in solid tumours [27–29]. In breast cancer cell lines, DNA methyl transferase inhibitors increased MHC-I expression and antigen presentation, leading to an increased T cell infiltration in mouse models of breast cancer [27]. This increase in MHC-I expression is thought to be due to reduced methylation of *HLA* genes [27], but also through demethylation of endogenous retrovirus (*ERV*) genes that trigger cytosolic sensing of dsRNA (double stranded RNA) [28]. *ERV*s are a relic of ancient infections that comprise 8% of the human genome [30]. These genes are silenced through hypermethylation but treatment with DNA methyl transferase inhibitors induced their expression, activating the dsRNA sensing pathway [27,28]. This pathway stimulated an interferon type I cellular response and NFκB (nuclear factor κB)-mediated activation of MHC-I expression.

#### Histone regulation

Histone modifications by trimethylation of lysine residues on histone 3 (H3K27me3) or deacetylation result in gene silencing and have been shown as mechanisms invoked by tumour cells to silence the APM [31,32]. An *in vitro* whole genome CRISPR/Cas9 screen in leukemia cell lines revealed EZH2 (enhancer of zest homolog 2) as a negative regulator of MHC-I, the master regulator of MHC-I transcription NRLC5 (nucleotide-binding domain and leucine-rich repeat caspase recruitment domain-containing 5), and TAP expression [33]. EZH2 catalyses the trimethylation of H3K27 leading to inhibition of transcription. Reversal of the H3K27me3 with an EZH2 inhibitor up-regulated MHC-I in leukemia as well as neuroblastoma and SCLC cells. In diffuse large B cell lymphoma (DLBCL) with *EZH2^Y641^* mutation, treatment with an EZH2 inhibitor reduced H3K27me3 in the promoter region of *NLRC5*, resulting in increased NLRC5 and MHC-I expression [34]. EZH2 inhibition also increased antigen presentation in head and neck squamous cell carcinoma cell lines, restoring sensitivity to anti-PD1 therapy in *in vivo* mouse model of head and neck cancer [35]. Histone deacetylase (HDAC) inhibitors have also demonstrated in vitro and in vivo efficacy in restoring MHC-I expression and immune control for various solid organ cancers [32,36], either as monotherapy or in combination with ICIs. Several human clinical trials are in progress examining their efficacy [37].

### Transcriptional modulation of MHC-I

#### IFNγ-dependent

Transcriptomic regulation of MHC-I is tightly controlled to elicit an appropriate immune response. The transcriptional transactivator NLRC5 is a critical regulator of MHC I expression. It forms a scaffold with DNA binding proteins RFX (regulatory factor X), CREB (cAMP responsive element binding protein 1), ATF1 (activating transcription factor 1) and NF-Y (nuclear factor Y) to form the CITA complex (class I transactivator) on the proximal promoter of *HLA* genes [38]. These regulatory elements and transcription factor/transactivator complex are also present on the promoter of other APM genes including *TAP1* and *B2M*. *In vivo* deletion of NLRC5 resulted in loss of MHC-I expression in mice, without altering MHC-II expression, demonstrating the critical role of NLRC5 in specifically controlling MHC-I expression. IFNγ is a key regulator of MHC-I expression through JAK1/2 (janus kinase 1/2)-STAT1 (signal transducer and activator of transcription 1) signalling and activation of NLRC5 expression. This transduction pathway also activates IRF1/2 (interferon regulatory factor 1/2) expression that binds the ISRE (interferon-stimulated response element) present in the proximal promoter of *HLA* genes. Alterations of these key transcription factors through genetic deletions or epigenetic modification results in loss of MHC-I expression. *NLRC5* loss has been described in several solid organ cancers [39,40]. Its absence abrogates MHC-I expression and CD8^+^ T cell mediated cytotoxic responses, thus conferring inferior patient survival [39]. Similar findings have been ascribed to the loss of *IRF1/2*, particularly in melanoma patients with ICI resistance [41]. Defects in IFNγ signalling through loss of *JAK1* or *JAK2* has also been associated with reduced MHC expression and resistance to ICIs [42,43]. DUX4 (double homeobox 4), a pre-implantation embryonic transcription factor normally silenced in somatic tissues, was found reactivated in many cancers. Its expression reduced MHC-I expression, likely through DUX4-mediated inhibition of JAK1 and STAT1 expression [44]. DUX4 overexpression was associated with resistance to immune checkpoint blockade in melanoma [44].

#### IFNγ-independent

Defects in IFNγ signalling is an important mechanism of primary and acquired resistance to ICIs [45–48]. Targeting the IFNγ pathway to increase MHC-I expression may therefore not represent an appropriate strategy to increase neoantigen presentation. Recent efforts have aimed to uncouple MHC-I expression from interferon signalling to increase MHC-I expression in tumours with defective IFNγ signalling [49]. NFκB is a known regulator of MHC-I expression through direct binding of p50/p65 subunits to NFκB response elements present in the enhancer region of *HLA-A* and *B* [50]. NFκB is activated by double-stranded RNA (dsRNA) sensors such as TLR3 (toll-like receptor) and PKR (serine/threonine kinase R) [51,52]. MHC-I, TAP1 and B2M expression were found to be up-regulated after treatment of melanoma cells with BO-112, an activator of dsRNA sensing and NF-κB signalling, restoring the cytotoxic activity of tumour-specific T cells [49]. Multiple agents can induce dsRNA sensors and could potentially be combined with ICIs to re-establish anti-tumour immunity.

#### Transcriptional regulations associated with oncogenic drivers

Aberrant activation of cell signalling pathways through oncogenic drivers can down-regulate MHC-I. An *in vitro* shRNA screen targeting 526 kinases identified the MAP kinase (MAPK) pathway, including downstream kinases MEK and ERK, as negative regulators of HLA-A expression [53]. Tumour cells with oncogenic activating mutations in the EGFR (epidermal growth factor receptor), ALK (anaplastic lymphoma kinase) or RET (rearranged during transfection) kinases were found to reduce MHC-I expression. Pharmacological inhibition of these kinases increased MHC-I cell surface expression, potentially increasing immune recognition [53,54].

The MYC family of proteins regulates transcription of ∼15% of the human genome [55]. Over-expression or dysregulation of the N-MYC and C-MYC oncoproteins is observed in up to 70% of human tumours and is associated with reduced immunosurveillance [56–58]. Mechanisms responsible for immune evasion of MYC-expressing tumour are starting to emerge with the observation that MYC prevented loading of dsRNA to TLR3 in pancreatic cancer cells, reducing NFκB signalling and MHC-I expression [58].

### Post transcriptional MHC-I regulation of

#### MicroRNAs (miRNAs)

miRNAs are a class of non-coding RNAs that are characterised by their short length (∼21–25 bp). They can bind to the 3′ untranslated region (UTR) of mRNA and inhibit their translation through mRNA degradation or translational repression. Binding sites for *mir-148a-3p* and *mir-125a-5p* were found in the 3′ untranslated region of *HLA-A*, *-B*, *-C* mRNA and *TAP2* mRNA respectively. Overexpression of *mir-148a-3p* reduced cell surface expression of MHC-I in colorectal and oesophageal cancer [59,60], while inhibition of *mir-148a-3p* restored MHC-I expression and increased T-cell mediated killing *in vitro* and *in vivo* [60]. miRNAs may be therapeutically targeted using complementary antisense RNAs (anti-miRs) packaged in lipid nanoparticles for optimal drug delivery of the oligonucleotides [61].

#### Long non-coding RNAs (lncRNAs)

Like miRNAs, lncRNAs are not translated into proteins. lncRNAs are >200 bp long that predominantly reside in nuclei. They are responsible for diverse processes that result in transcriptional and post-transcriptional regulation of gene expression. The oncogenic lncRNA *LINK-A* was recently found to be a negative regulator of MHC-I and B2M cell surface expression in triple negative breast cancer cells (TNBC), and a negative predictive biomarker in patients treated with ICIs [62]. *LINK-A* was shown to abrogate phosphorylation of the E3 ubiquitin ligase TRIM71, resulting in increased degradation of the MHC-I peptide loading complex. Other lncRNAs have been associated with positive regulation of MHC-I expression, such as *LINC02195* that positively correlated with MHC-I-related protein expression in head and neck squamous cell carcinomas cell lines and patient samples [63].

### Post translational regulation of MHC-I

Cancer cells may also evade immune recognition through post translational modification of MHC-I. ER-associated protein degradation (ERAD) constitutes a quality control system to eliminate misfolded or unassembled proteins from the ER [64]. Tumour cells exploit this pathway to induce degradation of the nascent MHC-I chain to hinder antigen presentation. Staphylococcal nuclease and tudor domain containing 1 (SND1), an oncoprotein overexpressed in solid tumours, guides the heavy chain of MHC-I to the ERAD, resulting in its dislodgement into the cytoplasm and subsequent degradation. Loss of SND1 increased MHC-I expression and cytotoxic T cell infiltration in *in vivo* models of melanoma and colorectal cancer, resulting in decreased tumour burden [65].

Increased turnover of the antigen loaded MHC-I is another mechanism by which tumour cells evade immune surveillance. Expression of the transmembrane protein MAL2 (myelin and lymphocyte protein 2) is associated with worst prognosis in TNBC cells [66]. Molecular analysis demonstrated that MAL2 promoted intracellular endocytosis of peptide bound MHC-I complexes through direct interactions with endosome-associated proteins [66]. Knockout of *MAL2* in patient-derived tumour organoid models resulted in enhanced CD8^+^ T cell-mediated cytotoxicity, thus making MAL2 a potential therapeutic target.

Tumours may also modify their cell membranes to sterically inhibit MHC-I interactions with CD8^+^ T cells. Specifically, high cell surface expression of glycosphingolipids by tumour cells impedes MHC-I and CD8^+^ T cell interaction [66]. Membrane expression of glycosphingolipids is modulated by the protease SPPL3 (signal peptide peptidase like 3). SPPL3 loss has been shown to be a negative prognostic biomarker in gliomas [67]. Reduced SPPL3 activity increased cell surface expression of glycosphingolipids, forming a shield preventing presentation of MHC-I-loaded peptides to CD8^+^ T cells. There is considerable interest in inhibiting glycosphingolipids synthesis using clinically approved inhibitors which have demonstrated in vitro efficacy in glioma cell lines [66].

Autophagy has been proposed as another mechanism utilised by tumour cells to reduce cell surface expression of MHC-I and avoid immune recognition [68,69]. Immunofluorescence analysis of human pancreatic ductal adenocarcinoma (PDAC) tumours and NSCLC cell lines demonstrated a preponderance for intracellular sequestration of MHC-I proteins in the autophagosomes and lysosomes [69]. Genetic inhibition of autophagy or pharmacological lysosomal inhibition resulted in increased total and cell surface expression of MHC-I, indicating a specific role for autophagy in the trafficking of MHC-I to the lysosome [69]. ATG4B (autophagy related 4B cysteine peptidase) is a cysteine protease that has an essential role in autophagosome formation. Inhibition of autophagy in genetically engineered murine PDAC cells expressing a dominant-negative form of ATG4B increased cell surface expression of MHC-I, tumour cell killing in *in vitro* co-culture assay with cytotoxic T cells and enhanced CD8^+^ T cell infiltration *in vivo* [68,69]. These tumours also responded more efficiently to ICI therapy than their wild-type counterparts [69]. These findings are particularly notable given the lack of efficacy using ICIs in clinical trials for patients with PDAC [70].

## MHC-II

### MHC-II function and processing pathway

Immuno-oncology research thus far has predominantly focussed on augmenting cytotoxic CD8^+^ T cell responses. However, there is increasing interest in harnessing CD4^+^ T helper cells to potentiate sustained anti-tumour immunity [71,72]. CD4^+^ T cells are activated by MHC-II-bound peptides. MHC-II molecules present exogenously derived peptides and have traditionally been associated with professional antigen presenting cells (APCs) such as dendritic cells, macrophages and B cells [73]. While tumour cells do not constitutively express MHC-II, IFNγ present in the tumour microenvironment can induce MHC-II in tumour cells (tsMHC-II). Indeed accumulating evidence now highlight a critical role for tsMHC-II towards the activation of CD4^+^ T cells [5]. CD4^+^ T helper cell differentiation is induced by the binding of a naïve CD4^+^ TCR to an MHC-II peptide complex combined with a second co-stimulatory signal where CD28 on CD4^+^ T cells binds to CD80/86 found on professional APCs. These T helper cells promote CD8^+^ T cell mediated responses and immunological memory [71,74]. Tumour cells do not express the classical co-stimulatory ligands CD80/86 [75]. However, they may utilise other cell-surface proteins to interact with CD28 on CD4^+^ T cells. Examples of these co-stimulatory molecules include OX40 and CD70, both found in solid cancers [76,77].

The presence of tsMHC-II is associated with increased CD4/CD8 tumour infiltrating lymphocytes, improved survival and responsiveness to ICIs [78–80]. Analysis of a cohort of melanoma patients treated with ICIs also revealed that the loss of tsMHC-II and MHC-I were not interdependent, suggesting that they may be independently regulated in cancer [81]. In a study of 5942 tumours, neoantigens that poorly bound to MHC-II were positively selected during cancer evolution. The degree of positive selection was even stronger than the association observed between MHC-I and its neoantigens [82]. These findings suggest that CD4^+^ T cell-mediated immunosurveillance may be a dominant mechanism for immune control of tumours.

**Figure 2.**
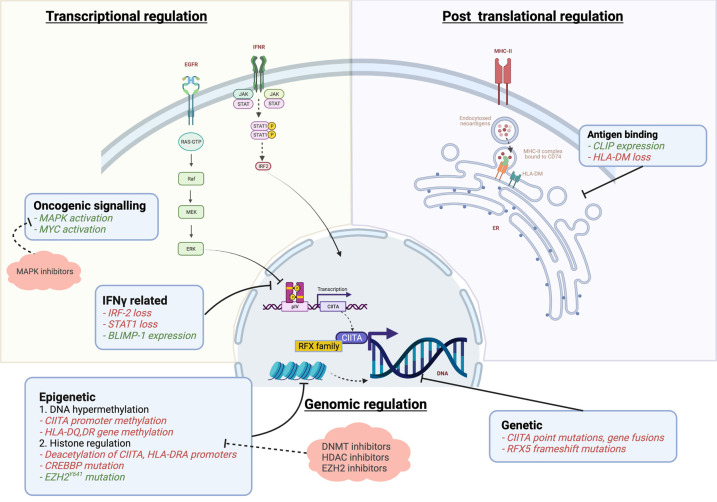
Mechanisms of down-regulation of MHC-II antigen presentation pathway by tumour cells and possible therapeutic targets. Green text: expression/over-expression negatively regulates antigen presentation. Red text: reduced/loss of expression negatively regulates antigen presentation.

Both MHC-I and MHC-II genes are highly polymorphic. However, MHC-II can bind a greater diversity of neoantigenic proteins. Their binding pocket can allow peptides of a longer length (>13 amino acid) and accommodates peptide side chains. The regulation of antigen processing and presentation by MHC-II in professional APCs has been reviewed elsewhere [73]. Here we focus on findings pertaining to the regulation of MHC II in non-professional antigen presenting tumour cells (Figure 2).

### Immune evasion through down-regulation of MHC-II in cancer

Little is known on the mechanisms driving the regulation of tsMHC-II. However, some studies are starting to emerge elucidating immune-evasion mechanisms associated with MHC-II complex down-regulation [83].

Expression of MHC-II is controlled by the transcriptional master regulator class II transactivator (CIITA) [84]. The CIITA complex is a scaffold of proteins that recruit activators, including RFX5, at transcriptional start sites of MHC-II related genes. They are a key component of MHC-II induction, though never binding DNA directly. The expression of CIITA is controlled by four promoters: promoters I (*pI*), II (*pII*), III (*pIII*), IV (*pIV*) [85]. Constitutive expression of CIITA in APCs is predominantly regulated by *pI* and *pIII*. The strongest inducer of CIITA in response to IFNγ stimulation is *pIV* [86]. Modulation of MHC-II expression in cancer has been associated with perturbed regulation of CIITA expression through genomic, epigenetic, transcriptional or post-translational mechanisms.

### Genetic mechanisms of MHC-II down-regulation

Genomic alterations in the *CIITA* gene, including point mutations and gene fusions have been observed in different types of lymphoid tumours [87,88]. Point mutations in *CIITA* or its promoter complex have also been observed in melanoma and microsatellite unstable (MSI-H) colorectal cancer (CRC)[89,90]. Frameshift mutations in the *RFX5* gene are also a common event in MSI-H CRC, occurring in approximately a quarter of cases [91]. These alterations resulted in reduced tsMHC-II expression and immunogenicity of tumour cells.

### Epigenetic silencing

#### DNA hypermethylation

DNA hypermethylation has been described at *CIITA*-promoter sites or directly affecting *MHC-II* genes. Hypermethylation of *CIITA-pIV* has been demonstrated in gastric cancer [92]. Hypermethylation of *HLA-DR* and *HLA-DQ* genes and absence of tsMHC-II expression have been associated with inferior survival in patients with oesophageal squamous cell carcinoma [93]. DNA methyltransferases, such as DNMT1 and DNMT3B, mediate these methylation effects. Their inhibition through genetic inactivation or pharmacological agents have been shown to induce MHC-II expression in colorectal and breast cancer cell lines [27,92].

#### Histone regulation

Histone acetylation promotes transcription of MHC-II related genes. B cell lymphoma cells with MHC-II expression were characterised by H3 and H4 acetylation at the *HLA-DRA* promoter compared with cell lines lacking MHC-II [94]. This process was shown to be induced by IFNγ [94]. In B cell lymphomas, inactivating mutation in the histone acetyl transferase CREBBP resulted in reduced MHC-II expression, further showing the importance of histone acetylation in MHC-II expression [95,96]. Histone de-acetylation at *CIITA* or *HLA-DRA* promoters has been observed *in vitro* in several solid organ and haematological malignancies, abrogating MHC-II expression [94,97,98]. Pre-clinical data support a role for HDAC inhibitors in up-regulating tsMHC-II expression through a CIITA-dependent mechanism [98,99].

Histone methylation driven by EZH2 has also been shown to regulate MHC-II expression in DLBCL where tumours with *EZH2^Y641^* mutation had low expression of MHC-I and II [34]. Treatment of these cell lines with an EZH2 inhibitor increased MHC II expression by reducing H3K27me3 on the *CIITA* promoter. Importantly this work not only provides a rationale for targeting EZH2 in combination with ICIs, but also identifies *EZH2* mutation as a biomarker to stratify patients who may respond to this combination therapy.

### Transcriptional modulation of MHC-II

Loss of interferon signalling can reduce MHC-II transcription. IRF2 has been shown to be a transcriptional activator of the *CIITA-pIV* promoter [100]. *IRF2* loss is described in several cancers and associated with attenuation of MHC-I and MHC-II expression [100,101]. Activation of the MAPK pathway also appears to be associated with reduced expression of MHC-II in NSCLC cell lines [102]. This effect was reversed using MEK inhibitors, indicating that inhibition of the MAPK pathway may increase tsMHC-II expression.

CIITA can also be inhibited by factors that competitively bind to E-box elements in the *CIITA-pIV* region, thus preventing transcription. The oncogenes L-MYC and N-MYC have been shown to bind this region in SCLC cell lines, resulting in loss of CIITA transcription [103]. Over-expression of the C-MYC oncogene in Burkitt's Lymphoma was also found to impair MHC-II antigen presentation through several mechanisms including reduced expression of the chaperone protein HLA-DM that regulates neoantigen binding to the MHC-II groove [104]. CIITA expression is also affected by loss of STAT1 and retinoblastoma tumour suppressor genes, as observed in SCLC, breast and thyroid carcinoma cell lines [105,106]. Conversely, BLIMP-1 (B lymphocyte-induced maturation protein I) acts as a developmentally conserved repressor of *CIITA* transcription and is associated with plasma cell differentiation in myeloma [107,108].

### Regulation of MHC-II antigen binding

The MHC-II complex is a heterodimer assembled in the ER with the chaperone protein CD74, also known as the invariant chain, to prevent loading of endogenous peptides. The MHC-II/CD74 complex is transported from the ER and fuses with acidic endosomes where exogenous peptide loading occurs. Cleavage of CD74 leaves the short fragment CLIP (class II-associated invariant peptide) blocking the peptide binding groove of MHC-II [109]. The chaperone protein HLA-DM releases CLIP for degradation and catalyses the binding of exogenous peptides to the MHC-II binding groove. Given that CLIP prevents peptide binding onto MHC-II complexes until it associates with HLA-DM, its expression is generally inversely proportional to HLA-DM [110]. Higher levels of CLIP have been associated with worse prognosis in acute myeloid leukemia [111]. In contrast, high expression of HLA-DM appears to portend improved survival in ovarian cancer [112]. These findings may relate to the impact of unhindered peptide/MHC-II binding towards establishing a robust anti-tumour response.

## Conclusions

Regulation of the APM in cancer is a critical mechanism that governs the anti-tumour immune response, ultimately determining survival outcomes for patients with cancer. Our review highlights the mechanisms of MHC-I/MHC-II regulation in tumour cells. Considerable studies have been undertaken to elucidate resistance mechanisms contributing to reduced immune visibility, particularly during the current era of immunotherapeutics. Yet more research is required to understand mechanisms of APM down-regulation that underpin resistance to current ICIs and discover novel regulators that may unleash anti-tumour immunity.

## Perspectives

- Despite the promise of long-term survival using immunotherapeutics in patients with otherwise incurable cancer, many do not respond to treatment due to immune evasion by tumour cells. This review outlines the mechanisms that tumour cells utilise to down-regulate neoantigen presentation to avoid immune recognition and highlights current strategies that may reactivate these pathways.
- Regulation of antigen presentation machinery in tumour cells may occur due to genomic, transcriptomic and post-translational modifications. Whilst most evidence to date focuses on elucidating mechanisms of MHC-I down-regulation, emerging research highlights the ability of tumour cells to express MHC-II and impact adaptive anti-tumour immunity.
- Ongoing research aims to identify novel mechanisms of neoantigen presentation regulation. Targeting these pathways with novel or repurposed drugs may enable immunotherapy to work for patients with otherwise limited treatment options.

## Open Access

Open access for this article was enabled by the participation of University of Melbourne in an all-inclusive *Read & Publish* agreement with Portland Press and the Biochemical Society under a transformative agreement with CAUL.
